# Phenolic Compounds and Antioxidant Activity of *Phalaenopsis* Orchid Hybrids

**DOI:** 10.3390/antiox5030031

**Published:** 2016-09-14

**Authors:** Truong Ngoc Minh, Do Tan Khang, Phung Thi Tuyen, Luong The Minh, La Hoang Anh, Nguyen Van Quan, Pham Thi Thu Ha, Nguyen Thanh Quan, Nguyen Phu Toan, Abdelnaser Abdelghany Elzaawely, Tran Dang Xuan

**Affiliations:** 1 Graduate School for International Development and Cooperation, Hiroshima University, Hiroshima 739-8529, Japan; minhtn689@gmail.com (T.N.M.); dtkhang@ctu.edu.vn (D.T.K.); phungtuyen@gmail.com (P.T.T.); ltminh87@gmail.com (L.T.M.); hoanganh6920@gmail.com (L.H.A.); nguyenquan26@gmail.com (N.V.Q.); phamthithuhabt@gmail.com (P.T.T.H.); quanagi@gmail.com (N.T.Q.); nguyenphutoan1983@gmail.com (N.P.T.); 2 Department of Agricultural Botany, Faculty of Agriculture, Tanta University, Tanta 31527, Egypt; elzaawely@agr.tanta.edu.eg

**Keywords:** *Phalaenopsis*, phenolic compounds, antioxidant activity, moth orchids

## Abstract

*Phalaenopsis* spp. is the most commercially and economically important orchid, but their plant parts are often left unused, which has caused environmental problems. To date, reports on phytochemical analyses were most available on endangered and medicinal orchids. The present study was conducted to determine the total phenolics, total flavonoids, and antioxidant activity of ethanol extracts prepared from leaves and roots of six commercial hybrid *Phalaenopsis* spp. Leaf extracts of “Chian Xen Queen” contained the highest total phenolics with a value of 11.52 ± 0.43 mg gallic acid equivalent per g dry weight and the highest total flavonoids (4.98 ± 0.27 mg rutin equivalent per g dry weight). The antioxidant activity of root extracts evaluated by DPPH (2,2-diphenyl-1-picrylhydrazyl) free radical scavenging assay and β-carotene bleaching method was higher than those of the leaf extracts. Eleven phenolic compounds were identified, namely, protocatechuic acid, *p*-hydroxybenzoic acid, vanillic acid, caffeic acid, syringic acid, vanillin, ferulic acid, sinapic acid, *p*-coumaric acid, benzoic acid, and ellagic acid. Ferulic, *p*-coumaric and sinapic acids were concentrated largely in the roots. The results suggested that the root extracts from hybrid *Phalaenopsis* spp. could be a potential source of natural antioxidants. This study also helps to reduce the amount of this orchid waste in industrial production, as its roots can be exploited for pharmaceutical purposes.

## 1. Introduction

Vegetables, spices, and herbs contain important natural substances such as antioxidants. Several studies have dealt with antioxidant activity to find new sources of natural antioxidants to be used in foods, cosmetics, medicine, and other purposes [1,2]. Antioxidants play an important role in health care to prevent and scavenge free radicals; alleviate chronic diseases and degenerative ailments such as cancer, autoimmune disorders, hypertension, atherosclerosis; and delay the ageing process [3,4,5,6,7,8,9]. Of all the plant secondary metabolites, phenolic compounds have been extensively studied and are commonly used as antioxidants for a wide range of applications [10]. Thus, investigation of new and safe antioxidants from natural sources has become very important for food and medicinal functions [11].

Orchids are the most species-rich of all angiosperm families (estimated > 25,000 species) with a wide diversity of epiphytic and terrestrial growth forms and have successfully colonized most habitats on earth [12]. To date, only very few studies have been conducted on orchids as a potential source of antioxidants. In 2007, Ling and Subramaniam [13] reported the chemical composition, biochemical analyses, and antioxidant activity of the leaves and flowers of *Phalaenopsis* (*Phal.*) *violacea* orchid. On the other hand, the chemical composition and antiradical properties of *Dendrobium speciosum* have been assessed [14]. It is, therefore, believed that orchids are potential sources of antioxidants. Hence, *Phalaenopsis* species are considered in this study.

*Phalaenopsis* species, belonging to family Orchidaceae, also known as the moth orchid, are the most common and commercially important orchids due to its beautiful flowers and long-time blooming. These orchids are fleshy and distichous evergreen leaves that make it both attractive and distinctive in the floriculture industry. They are marketed as potted plants and cut flowers leading to their tremendous production over years. Currently, many countries are developing the production of these orchids because of its benefit. In 2009, Thailand exported 211 genera of orchid plants, among them, the *Phal.* plant was important with the market share of 25.5% of total quantity [15]. Chien et al. [16] reported that, in 2012, Taiwan is the leading country of exporting *Phal.*, providing the largest share (69%) of total 164.70 million US dollars. Additionally, in 2014, 80 million products of *Phal.* were manufactured from tissue culture in this country [17]. However, after flowering one or two times, the plant will become very weak and become waste [16]. Therefore, this study aimed to utilize the orchid waste and assess its antioxidant potential. The leaves and roots of six different hybrid *Phal.* spp. were utilized and investigated in this study.

## 2. Experimental Section

### 2.1. Chemicals

All the analytical-grade chemicals used in this study were purchased from KANTO Chemical, Tokyo, Japan.

### 2.2. Plant Material

Six different hybrids of *Phal.* spp. were provided by the Kurousu Orchid Company, Saitama Prefecture, Japan in February 2014. The names and abbreviations of the six commercial *Phal*. hybrids are presented in Table 1.

### 2.3. Sample Preparation

Leaves and roots of each orchid species were separately cut into small pieces and dried in an oven at 30 °C. After drying, the samples were powdered using a kitchen grinder.

### 2.4. Preparation of Ethanol Crude Extracts (Free Phenolics)

The powdered leaves and roots of each hybrid (0.5 g) were separately steeped in 50 mL ethanol for 24 h at room temperature. The extracts were then filtered and concentrated separately under vacuum at 40 °C using a rotary evaporator (SB-350-EYELA, Tokyo Rikakikai Co., Ltd., Tokyo, Japan). The dried crude ethanol extracts, considered free phenolic extracts, were prepared from leaves or roots. They were separately dissolved in methanol and kept in a refrigerator for the following steps [18].

### 2.5. Preparation of Ethyl Acetate Conjugate Crude Extracts (Conjugate Phenolics)

The leaf and the root residues after ethanol extraction were mixed separately with 40 mL of 4 M NaOH and constantly stirred at 60 °C for 4 h. The hydrolyzed solution was centrifuged for 10 min at 5000 rpm, and the supernatant was filtered. The resulting solution was acidified to pH 1.5 with 6 M HCl. Subsequently, it was centrifuged for 10 min at 5000 rpm and filtered afterward. The filtrates were extracted with 40 mL of ethyl acetate. The extracts were separately evaporated to dryness by a rotary evaporator at 30 °C and discretely dissolved in methanol and kept at 4 °C for further analyses [19].

### 2.6. Estimation of Phenolic Contents

Phenolic contents of ethanol crude extracts (free phenolics) or ethyl acetate crude extracts (conjugate phenolics), prepared from leaves and roots were calculated using Folin-Ciocalteu method, described by Elzaawely and Tawata [20]. Two hundred µL of each sample (0.5 mg/L) was mixed with 1.0 mL of Folin-Ciocalteu’s reagent (10%) and 0.8 mL of sodium carbonate (7.5%), respectively. The solutions were mixed and allowed to stand for 30 min. The mixture was then measured at 765 nm using a HACH DR/4000U spectrophotometer (HACH Company, Loveland, CO, USA). The total phenolic contents were expressed as mg gallic acid equivalents (GAE) per g dry weight (DW).

### 2.7. Estimation of Flavonoid Contents

The total flavonoids were determined according to the method described by Djeridane et al. [21]. One mL of extract (0.5 mg/mL) was mixed with 1 mL aluminum chloride (2%). The mixture was stirred and kept at room temperature for 15 min. The absorbance was measured at 430 nm using a HACH DR/4000U spectrophotometer. Total flavonoids were reported as mg rutin equivalents (RE) per g dry weight (DW).

### 2.8. Antioxidant Activity

#### 2.8.1. DPPH Scavenging Assay

The DPPH free radical scavenging assay described by Elzaawely et al. [19] was used to evaluate the antioxidant capacity of the six orchid hybrids. The mixture consisted of 0.5 mL sample extracts, 0.25 mL of 0.5 mM DPPH, and 0.5 mL of 0.1 M acetate buffer (pH 5.5). The mixture was kept in the dark at room temperature for 30 min. The absorbance was measured at 517 nm. BHT was used as a positive reference while methanol was used as a control. Radical scavenging activity was expressed as the inhibition percentage and was calculated using the formula: % radical scavenging activity = [(A_control_ − A_test_)/A_control_] ×100.

A_control_ corresponds to the absorbance of the control and A_test_ corresponds to the absorbance of the test extract. The IC_50_ value was also calculated using % radical scavenging activity. Lower IC_50_ values indicate higher antioxidant activity.

#### 2.8.2. β-Carotene Bleaching Method

Antioxidant activity was evaluated according to the β-carotene bleaching method described by Siddhuraju and Becker [22]. β-Carotene (2.0 mg) was dissolved in 10 mL chloroform and 1 mL of this solution was mixed with 20 µL linoleic acid and 200 mg Tween-40. The chloroform was evaporated under vacuum at 45 °C, then added by 50 mL oxygenated water. Successively, the mixture was vigorously shaken until an emulsion formed. A volume of 120 mL of the methanolic extract and 120 mL BHT (1000 ppm) were mixed with 1 mL of the emulsion in a test tube, which was then incubated at 50 °C. An equal amount of methanol was used for negative control. The absorbance of the mixture was measured using a HACH DR/4000U spectrophotometer at 492 nm. All samples were measured at zero time and every 15 min up to 180 min. Percentage of lipid peroxidation inhibition (LPI) was calculated using the following equation [23]: % LPI = A_1_/A_0_ × 100.

Where A_0_ is the absorbance value measured at zero time for the test sample, while A_1_ is the corresponding absorbance value measured after incubation for 180 min.

### 2.9. Quantification of Phenolic Compounds by HPLC

HPLC was used to determine both free and conjugate phenolics in different samples as described by Xuan et al. [20]. Accordingly, the extracts were separately filtered using 0.2 µm filter (KANTO chemical, Tokyo, Japan), then injected into the HPLC (JASCO PU-2089 Plus, JASCO Corporation, Tokyo, Japan, column J-Pak Symphonia C18 110A (4.6 mmØ × 15 mm), solvent system: (A) 0.1% of acetic acid, (B) 100% methanol, gradient program: 5–10 min, 5%–20% (A); 10–30 min, 20%–80% (A); 30–40 min, 80%–100% (A), wavelength: 254 nm and flow rate: 1.0 mL/min). Concentrations of phenolic compounds in the samples were calculated by comparing peak areas with the standards.

### 2.10. Statistical Analysis

Data were analyzed using ANOVA with significant difference determined at a confidence level of *p* < 0.05.

## 3. Results

### 3.1. Antioxidant Activity, Phenolic Contents, and Lipid Peroxidation Inhibition of Phal. Hybrid Extracts

Phenolic compounds are one of the major chemical classes of plants’ secondary metabolites. They play an important role in the defense of plants against pathogens, diseases, parasites, and predators [24]. They involve in a number of physiological mechanisms such as antioxidant activity. They also play an important role in stabilizing lipid peroxidation [25]. The amount of phenolics produced by a plant depends on several factors such as temperature, UV-light, nutrition available to the plant, and genetic factors [13].

From Table 2, SYV3 exhibited the highest radical scavenging activity (lowest IC_50_ values) in both free and conjugate forms. This result is in agreement with its phenolic contents in both extracts. It is also important to note that the roots of SYV3 gave the strongest radical scavenging activity among all samples used. Comparing the roots and the leaves of all samples, the former contain more phenolics than the latter and they showed higher free radical scavenging activity in the order SYV3 > GFSV > FBD > YGL > SH > CXQ.

**Figure 1:**
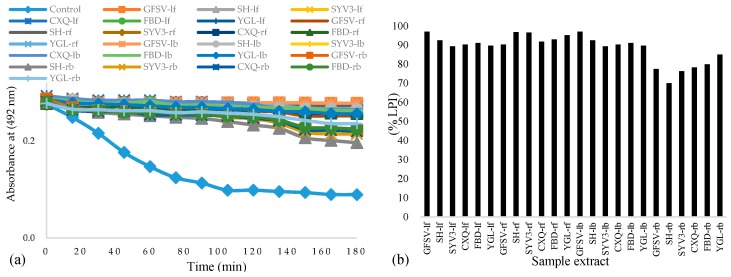
Antioxidant activity of the extracts measured by β-carotene bleaching method (**a**) and their lipid peroxidation inhibition (% LPI) (**b**). Notes: lf-leaves free phenolics; rf-roots free phenolics; lb-leaves conjugate phenolics; rb-roots conjugate phenolics.

It has been reported that the phenolics in plants possess strong antioxidant activity and levels of the antioxidant activity are proportional to the concentrations [26]. In the presence of antioxidants, the free radical activity of linoleic acid is hindered, thus preventing it from attacking the polyunsaturated β-carotene [23]. The antioxidant activity and the percentage lipid peroxidation inhibition (% LPI) results of the different extracts are presented in Figure 1. It is clear that the presence of antioxidants in different extracts prepared from *Phal*. leaves or roots reduced the oxidation of ß*-*carotene. The LPI values of free phenolic extracts ranged from were 89.4% to 97.0%, while those of conjugate phenolic extracts were 70.1% to 97.0%.

### 3.2. Total Flavonoids Contents

**Figure 2:**
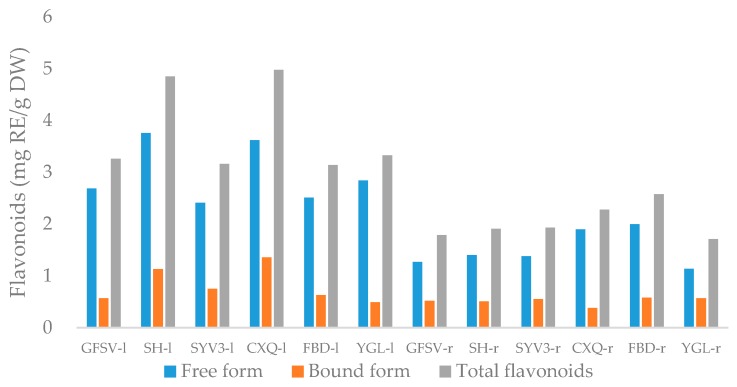
Total flavonoid contents of *Phal.* hybrid samples. Notes: l-leaves; r-roots.

Flavonoids have the ability to repress free radicals, reduce their levels in the body, and increase antioxidant defense activity [24]. Total flavonoids in six *Phal*. hybrids ranged from 1.71 ± 0.06 to 4.98 ± 0.27 mg RE/g DW (Figure 2). Among all extracts, a significantly high amount was found in leaf extracts of CXQ and SH with values 4.98 and 4.85 mg RE/g DW, respectively; while root extracts of YGL had the lowest amount (1.71 mg RE/g DW).

The results indicate that phenolic compounds are mostly found in the conjugate form, while most of the flavonoids are in the free form.

### 3.3. HPLC Quantification

**Figure 3:**
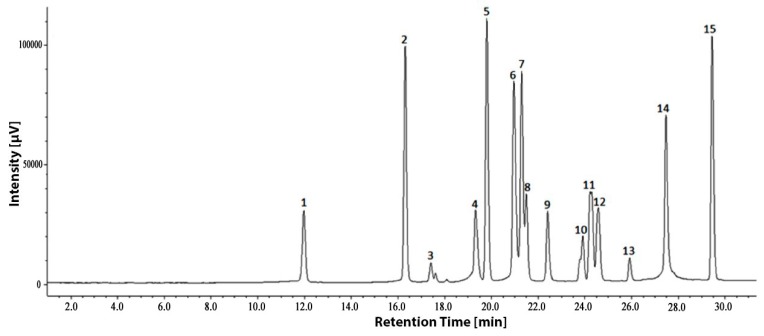
HPLC chromatogram (at 254 nm) of phenolic compounds used as standards. 1 = gallic acid (GA); 2 = protocatechuic acid (PA); 3 = catechol (C); 4 = chlorogenic acid (CHA); 5 = *p*-hydroxybenzoic acid (*p-*HBA); 6 = vanillic acid (VA); 7 = caffeic acid (CFA); 8 = syringic acid (SYA); 9 = vanillin (V); 10 = ferulic acid (FA); 11 = sinapic acid (SIA); 12 = *p*-coumaric acid (*p-*CA); 13 = benzoic acid (BA); 14 = ellagic acid (EA); 15 = cinnamic acid (CA).

From above results, the free and conjugate phenolics of leaves and roots of *Phal*. hybrids accounted for high concentration and showed strong antioxidant activity, therefore, they were analyzed by HPLC for identification and quantification of individual phenolics. The HPLC chromatogram of the 15 phenolic standards used in this study is presented in Figure 3.

**Figure 4:**
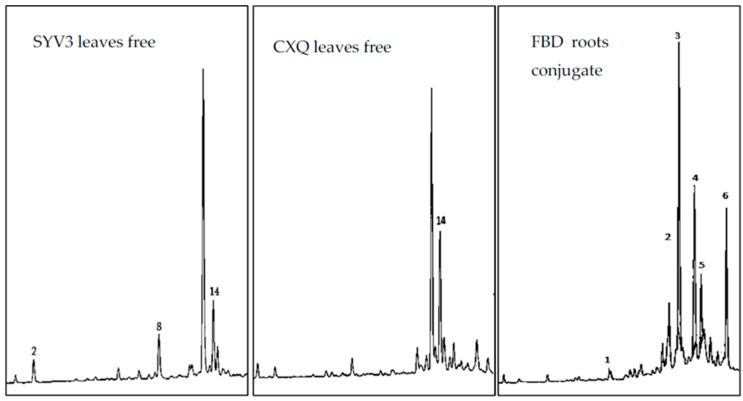
HPLC fingerprints of some samples (at 254 nm).

Of the 15 standard phenolic compounds used, seven compounds including protocatechuic acid (PA), syringic acid (SYA), ferulic acid (FA), sinapic acid (SIA), *p*-coumaric acid (*p-*CA), benzoic acid (BA), and ellagic acid (EA) were detected in the free phenolic extracts. PA, SYA, FA, SIA, BA, and EA were found in leaf extracts, while FA, *p*-CA, and EA were identified in root extracts (Figure 4, Table 3). In all conjugate phenolic extracts, EA was the most common and the leaf extracts of CXQ hybrid contained the highest amount of EA (346.3 µg/g DW).

On the other hand, 11 phenolic compounds including PA, *p*-HBA, VA, CA, SYA, V, FA, SIA, *p*-CA, BA, and EA were identified in the conjugate phenolic extracts and they were found mostly in root extracts (Figure 4, Table 4 and Table 5). Among these phenolics, FA, *p-*CA, and SIA were the predominant in the roots. The highest concentration of FA (432.68 µg/g DW) was detected in the conjugate phenolic leaves extracts of YGL hybrid, while the highest concentration of SIA (2232.81 µg/g DW) and *p-*CA (767.81 µg/g DW) were detected in the roots extracts of SYV3 and SH hybrids, respectively. It was previously reported that phenolic acids such as ferulic, sinapic, *p*-coumaric, and ellagic acids possessed strong antioxidant activity [26,27,28]. Ellagic acid has been tested on many biological activities consisting of anti-inflammation, anti-proliferation, anti-angiogenesis, anticarcinogenesis, antimutagenensis, anti-cancer, and antiradical [29].

Phenolic acids are usually found in plants in bound form as esters and glycosides, therefore, alkaline, acidic or enzymatic hydrolysis is needed to release them from its conjugate state [18]. In this study, alkaline hydrolysis with 4 M NaOH at 60 °C was performed to extract conjugated phenolics. The increase of antioxidant activity of the tested extracts after alkaline hydrolysis may be due to the release of bound phenolics from their conjugates [28].

## 4. Discussion

Orchiaeae is one of the world’s largest families of flowering plants of angiosperms with their diverse shapes, forms, and colors. The potted plants and cut flowers of orchids are marketed and lead to a tremendous production over years [24,30]. Except for important economic value, the search for phytochemicals which may be exploited for herbal drug preparations on commercial orchids has been conducted sporadically [24,31], whilst extensive reports were on medicinal and endangered endemic orchid species [32,33,34,35,36,37,38,39,40,41,42,43,44]. The presence of various active compounds including dendrobine, moscatilin, gigantol, denbinobine, nobiline, and dendrophenol in the stems and leaves of the commercial *Dendrobium nobile* Lind, has greatly increased its medicinal importance [45,46,47]. These compounds were reported to have strong antimutagenic properties, consisting of anti-carcinogenic effects against lung carcinoma, ovary adenocarcinoma, and promyelocytic leukemia [48]. Moin et al., [31] reported the presence of phenols, flavonoids, alkaloids, phlobatannins, terpenoids, glycosides, tannins, saponins, and phytosterols in different extracting solvents including petroleum ether, ethyl acetate, methanol, and distilled water of *Coelogyne stricta* leaf extracts, an ornamentally and medicinally important orchid in Asia. In *Dendrobium pandurantum*, the presence of steroids, triterpenoids, alkaloids, tannins, phenols, and flavonoids were found [43]. In *Cymbidium aloifolium*, flavonoids, phenols, quinones, coumarins, saponins, alkaloids, carbonhydrates, tannins, cardiac glycosides, and osalates were found in different parts of the plant (leaves, roots, seeds, and capsule covers) [33]. In other orchid species including *Rhynchostylis retusa* [41], *Eria pseudoclavicaulis* [31], *Vanda tessellate* [39], *Monodora tenuifolia* [38], *Dactylorhiza chuhensis* [36], *Dendrobium officinale* [35], *Bauhinia variegate* [34], phytochemicals belonging to many chemical classes were identified, including alkaloids, coumarins, tannins, phenols, carbohydrates, terpenoids, steroids, and flavonoids. In *Dendrobium ovatum*, a threatened medicinal orchid, a bibenzyl compound (stilbene or moscatilin) was identified and quantified [37]. This natural product derived from this orchid species has been known to possess anti-mutagenic and anti-cancer properties [37]. In addition, Maridass et al., [42] screened the phytochemical profile of 61 orchid species in the Tiruneleveli Hills of South India and reported the existence of flavonoids, cyanogenic glycosides, terpenoids, and tannins. Other important phytochemicals were found in orchids such as stilebnoids, anthocyanins, triterpenoids, orchinol, hircinol, cypripedin, bibenzyl derivatives, phenanthrenes, jibantine, ndemin, and loroglossin, in either plant parts or entire plants [49,50,51,52,53,54,55].

The pharmaceutical uses of orchids such as anti-rheumatic, anti-inflammatory, antiviral, anti-carcinogenic, anticonvulsive, diuretic, neutroprotective, relaxation, anti-aging, wound healing, hypoglycemic, antitumor, anti-cancer, antimicrobial, antibacterial, antioxidant, anti-diarrheal, were reported [31,32,36,39,56,57,58]. Number of orchid species have been used as traditional medicines in Asia. Of them, different species of *Dendrobium* are used in Taiwan, Korea, and Japan for treatments of stomach ache, night sweats, and kidney strengthening. The tuber of *Bletilla striata* has been used for curing pneumonorrhagia and pneumonophthisis. The medicine prepared from these tubers are useful to treat tuberculosis, hemopysis, gastrisis, and duodenal ulcers, as well as bleeding and cracked skin on the feet and hands. It was reported as medicine for lung consolidation, treatment of pus, boils, abscesses, malignant swellings, ulcers, and breast cancer [54]. The use of orchids as traditional medicine was found in Asia, Africa, the Middle East, America, and Europe [59,60,61]. Other medicinal properties of orchids were reported such as tonic in hysteria, spasm, madness, and epilepsy, treatments of rheumatism, tuberculosis, body ache, eczema, headache and fever, aphrodisiac, and cardiac, respiratory, and nervous disorders [57].

Although many studies on orchids have been conducted so far, and many phytochemicals and pharmaceutical properties were reported, as mentioned above, most of work concentrated on medicinal and endangered orchid species. This study is the initial step to observe those plant parts, especially the roots of the importantly commercial *Phal.* contained rich antioxidants that should be exploited. This study did not aim to search novel constituents in the *Phal.* hybrids, but examined the utilization of its plant waste after production of commercial flowers. It was reported in Thailand, where an extensive quantity of orchids is produced, consumed, and exported, the leaf of unused orchid waste has caused environmental concerns [62]. Orchids have played an important role in economy of many countries, but its waste has caused many problems for human health [62,63]. Findings of this study suggest that the quantity of *Phal.* waste can be reduced when its plant parts can be exploited for medicinal purposes as the roots have been revealed to possess rich antioxidants. Total flavonoids, total phenols, and a number of phenolic acids were detected and quantified in this study, and showed relevance to antioxidant activity which are evidence to examine the potential of using this orchid species as herbal medicines. The next step of searching for novel bioactive compounds in *Phal.* is required, however it will need further complicated extracting processes and modern analytical instruments to determine the chemical structures of these potent compounds, such as NMR (nuclear magnetic resonance), IR (infrared spectroscopy), LC-MS (liquid chromatography-mass spectrometry, and GC-MS (gas chromatography–mass spectrometry). It has been estimated that about 400,000 secondary metabolites from plants may exist, but only about 2%–3% of these compounds have been isolated and identified [64]. In higher plants, the bioactive compounds are often in complicated chemical structures, which require laborious isolation and identification and costly in chemical synthesis to develop novel drugs derived from those plants. Therefore, for natural products derived from plants, the synthesis of derivatives from known compounds with simple chemical structures and testing for their novel biological activities is more promising, economic, and efficient for the development of novel pharmaceuticals.

## 5. Conclusions

This study indicated that the extracts prepared from leaves and roots of six *Phal.* hybrids contain high amounts of phenolic compounds and exhibit strong antioxidant activities. Ferulic acid, *p*-coumaric acid, and sinapic acid are concentrated largely in the roots in comparison with the leaves. Hence, the root extracts of *Phal.* orchid hybrids may be used as a potential source of natural antioxidants at a low cost.

## Figures and Tables

**Table 1:** Names and abbreviations of *Phal.* hybrids.

Name	Abbreviation
Green Field Sweet Valentine “Montclair”	GFSV
Sakura Hime	SH
Sogo Yukidian “V3”	SYV3
Chian Xen Queen	CXQ
Fusheng‘s Bridal Dress “Meidarland”	FBD
Younghome Golden Leopard “Peachy”	YGL

**Table 2:** DPPH radical scavenging activity in terms of IC_50_ values and phenolic contents of *Phal.* hybrid extracts.

Sample	DPPH IC_50_ (mg/mL)		Phenolics (mg GAE/g DW)	
	Free Form	Conjugate Form	Free Form	Conjugate Form
Leaves				
GFSV	> 4	0.534 ± 0.004 ^b^	1.65 ± 0.06 ^c,d^	4.57 ± 0.09 ^g^
SH	> 4	0.494 ± 0.016 ^c^	1.98 ± 0.03 ^b,c^	6.96 ± 0.10 ^d^
SYV3	> 4	0.430 ± 0.004 ^d^	2.29 ± 0.05 ^b,c^	5.50 ± 0.12 ^f^
CXQ	> 4	0.435 ± 0.008 ^d^	2.97 ± 0.45 ^b,c^	8.55 ± 0.02 ^a^
FBD	> 4	0.536 ± 0.000 ^b^	1.91 ± 0.12 ^b,c^	5.15 ± 0.06 ^f^
YGL	> 4	0.645 ± 0.003 ^a^	0.55 ± 0.00 ^d^	4.76 ± 0.14 ^d^
Roots				
GFSV	0.362 ± 0.021 ^c^	0.087 ± 0.000 ^e^	1.55 ± 0.32 ^c,d^	5.87 ± 0.02 ^e^
GFSV	0.427 ± 0.008 ^b^	0.092 ± 0.000 ^e^	2.34 ± 0.08 ^b,c^	6.91 ± 0.00 ^d^
SYV3	0.348 ± 0.001 ^c^	0.089 ± 0.000 ^e^	3.78 ± 0.44 ^a^	7.55 ± 0.10 ^b^
CXQ	0.715 ± 0.022 ^a^	0.100 ± 0.000 ^e^	1.44 ± 0.03 ^c,d^	7.14 ± 0.10 ^c,d^
FBD	0.364 ± 0.000 ^c^	0.098 ± 0.000 ^e^	4.01 ± 0.76 ^a^	6.91 ± 0.13 ^d^
YGL	0.379 ± 0.010 ^b,c^	0.092 ± 0.000 ^e^	3.61 ± 0.29 ^a^	7.42 ± 0.09 ^b,c^
BHT	0.019 ± 0.001		-	

Values are means of three replications ± SD; Means with the same letter in each column are not significantly different (*p* < 0.05); -: not detected

**Table 3:** Phenolic compounds of free form extracts of *Phal.* hybrids.

Sample	Contents of Phenolic Compounds (µg/g DW)						
	PA	SYA	FA	SI	*p*-CA	BA	EA
Leaves							
GFSV	133.65 ± 0.08 ^b^	-	-	-	-	-	38.34 ± 1.10 ^c^
SH	-	-	-	274.24 ± 9.85	-	178.60 ± 2.49	40.07 ± 1.89 ^c^
SYV3	147.09 ± 0.12 ^a^	99.18 ± 0.67	-	-	-	-	119.21 ± 0.54 ^b^
CXQ	-	-	-	-	-	-	346.30 ± 14.38 ^a^
FBD	-	-	216.05 ± 0.19	-	-	-	-
YGL	-	-	-	-	-	-	57.16 ± 1.15 ^c^
Roots							
GFSV	-	-	-	-	-	-	-
SH	-	-	-	-	-	-	118.20 ± 2.24 ^b^
SYV3	-	-	-	-	272.00 ± 2.83	-	-
CXQ	-	-	-	-	-	-	-
FBD	-	-	-	-	-	-	-
YGL	-	-	-	-	236.94 ± 19.62	-	-
					ns		

Values are means of three replications ± SD (standard deviation); Values with no letter in common in each column are not significantly different (*p* < 0.05); **-**: not detected; ns: not significantly different (*p* < 0.05); PA: protocatechuic acid; SYA: syringic acid; FA: ferulic acid; SIA: sinapic acid; *p-*CA: *p*-coumaric acid; BA: benzoic acid; EA: ellagic acid.

**Table 4:** Phenolic compounds of conjugate extracts of *Phal.* hybrids base on: PA, *p*-BHA, VA, CA, SYA, and V.

Sample	Contents of Phenolic Compounds (µg/g DW)					
	PA	*p*-HBA	VA	CA	SYA	V
Leaves						
GFSV	-	-	-	-	-	5.07 ± 1.07 ^c^
SH	122.31 ± 0.24 ^b^	77.44 ± 0.10	6.78 ± 1.72 ^d^	-	93.73 ± 3.47 ^c^	-
SYV3	-	-	-	-	-	-
CXQ	-	-	18.93 ± 3.78 ^d^	-	-	-
FBD	-	-	-	-	-	-
YGL	-	-	-	-	104.97 ± 1.04 ^b,c^	-
Roots						
GFSV	-	86.50 ± 4.22	85.25 ± 19.29 ^a,b,c^	-	125.54 ± 5.30 ^a^	119.15 ± 27.48 ^b^
SH	-	-	69.53 ± 0.21 ^c^	123.62 ± 23.66	-	-
SYV3	-	-	-	-	-	-
CXQ	-	-	107.24 ± 8.93 ^a,b^	-	106.18 ± 3.93 ^b,c^	113.31 ± 3.13 ^b^
FBD	-	-	77.99 ± 1.95 ^b,c^	-	109.81 ± 2.67 ^b^	97.58 ± 7.15 ^b^
YGL	184.24 ± 1.24 ^a^	-	117.84 ± 1.06 a	-	117.08 ± 1.82 ^a,b^	185.23 ± 1.52 ^a^

Values are means of three replications ± SD. Values with no letter in common in each column are not significantly different (*p* < 0.05); **-**: not detected; ns: not significantly different (*p* < 0.05); PA: protocatechuic acid; *p*-HBA: *p*-hydroxybenzoic acid; VA: vanillic acid; CA: caffeic acid; SYA: syringic acid; V: vanillin.

**Table 5:** Phenolic compounds of conjugate extracts of *Phal.* hybrids base on: FA, SIA, *p*-CA, BA, and EA.

Sample	Contents of Phenolic Compounds (µg/g DW)				
	FA	SIA	*p*-CA	BA	EA
Leaves					
GFSV	383.63 ± 22.21 ^a^	-	-	-	-
SH	414.31 ± 17.69 ^a^	1141.65 ± 67.00 ^c^	-	-	53.80 ± 6.49 ^a,b^
SYV3	414.95 ± 12.08 ^a^	709.15 ± 15.71 ^d^	232.31 ± 9.32 ^c^	-	31.51 ± 0.21 ^c^
CXQ	384.75 ± 6.19 ^a^	-	-	-	-
FBD	-	-	422.94 ± 70.13 ^b^	-	-
YGL	432.68 ± 19.03 ^a^	-	313.29 ± 28.47 ^b,c^	-	60.71 ± 7.37 ^a^
Roots					
GFSV	173.77 ± 4.67 ^c^	-	-	340.54 ± 106.00	-
SH	192.41 ± 3.44b ^c^	-	767.81 ± 20.67 ^a^	-	35.01 ± 5.56 ^b,c^
SYV3	221.61 ± 3.30b ^c^	2232.81 ± 29.71 ^a^	288.02 ± 9.31 ^c^	242.47 ± 10.65	46.99 ± 0.29 ^a,b,c^
CXQ	224.14 ± 13.29 ^b^	1639.24 ± 42.70 ^b^	298.20 ± 11.29 ^c^	268.62 ± 41.34	-
FBD	-	-	289.51 ± 0.64 ^c^	139.86 ± 50.62	36.23 ± 4.63 ^b,c^
YGL	227.80 ± 4.73 ^b^	1800.92 ± 39.45 ^b^	274.31 ± 2.12 ^c^	262.22 ± 13.36	-

Values are means of three replications ± SD. Values with no letter in common in each column are not significantly different (*p* < 0.05); **-**: not detected; ns: not significantly different (*p* < 0.05); FA: ferulic acid; SIA: sinapic acid; *p*-CA: *p*-coumaric acid; BA: benzoic acid; EA: ellagic acid.
